# Supplementary material for: RNA-guided RNA silencing by an Asgard archaeal Argonaute
Source: Nat Commun. 2024 Jun 29;15:5499. doi: 10.1038/s41467-024-49452-1 (PMC11217426; doi:10.1038/s41467-024-49452-1)
Supplement: Supplementary file 1 — Supplementary Information [file 41467_2024_49452_MOESM1_ESM.pdf]

Table S1: Guides and Targets used for in vitro cleavage assays.

Name	5’ modification	Sequence	Note
ogDS001	Phosphate group	TGAGGTAGTAGGTTGTATAGT	ssDNA guide
ogDS003	Hydroxyl group (none)	TGAGGTAGTAGGTTGTATAGT	ssDNA guide
ogDS002	Phosphate group	UGAGGUAGUAGGUUGUAUAGU	ssRNA guide
oBK458	Hydroxyl group (none)	UGAGGUAGUAGGUUGUAUAGU	ssRNA guide
oDS401	Cy5	AAACGACGGCCAGTGCCAAGCTTACTATACAACCTACTACCTCAT	ssDNA target
oDS403	Cy5	AAACGACGGCCAGUGCCAAGCUUACUAUACAACCUACUACCUCAU	ssRNA target

Table S2. Cryo-EM data collection, refinement and validation statistics

HrAgo1-guide RNA complex (EMD-18878) (PDB 8R3Z)	
**Data collection and processing**	
Magnification	130,000
Voltage (kV)	300
Electron exposure (e–/Å^2)	56.81
Defocus range (μm)	-1.0 to -2.4 (0.2 steps)
Pixel size (Å)	0.325
Symmetry imposed	C1
Initial particle images (no.)	8,161,440
Final particle images (no.)	283,659
Map resolution (Å)	3.4
FSC threshold	0.143
Map resolution range (Å)	3.0-5.5
**Refinement**	
Initial model used (PDB code)	Alphafold2
Model resolution (Å)	3.4
FSC threshold	0.143
Model resolution range (Å)	3.2-3.8
Map sharpening *B* factor (Å^2)	-123.1
Model composition	
Non-hydrogen atoms	6389
Protein residues	755
Nucleotide residues	6
Ligands	MG: 1
B factors (Å^2) min/max/mean	
Protein	57.80/253.73/152.50
Nucleotide	145.66/381.92/258.44
Ligand	140.84/140.84/140.84
R.m.s. deviations	
Bond lengths (Å)	0.004 (0)
Bond angles (°)	0.653 (3)
Validation	
MolProbity score	1.89
Clashscore	14.42
Poor rotamers (%)	0.29
Ramachandran plot	
Favored (%)	96.5
Allowed (%)	3.5
Disallowed (%)	0

Table S3. Sequences used to prepare guides and targets for single-molecule experiments

Name	Sequence (5’ --> 3’)
Let7a RNA guide N3	[phos]UGAGUAUU(5-LC-N-U)UUUUUUUUUUUUU
Let7a RNA guide N4	[phos]UGAGGAUU(5-LC-N-U)UUUUUUUUUUUUU
Let7a RNA guide N5	[phos]UGAGGUUU(5-LC-N-U)UUUUUUUUUUUUU
Let7a RNA guide N6	[phos]UGAGGUAU(5-LC-N-U)UUUUUUUUUUUUU
Let7a RNA guide N7	[phos]UGAGGUAGA(5-LC-N-U)UUUUUUUUUUUU
Let7a RNA guide N8	[phos]UGAGGUAG(5-LC-N-U)UUUUUUUUUUUUU
Let7a RNA guide N15	[phos]UGAGGUAG(5-LC-N-U)AGGUUGUUUUUUU
Let7a RNA guide N19	[phos]UGAGGUAG(5-LC-N-U)AGGUUGUAUAGUU
Let7a RNA target N6	UUUUUUUUUU(5-LC-N-U)UUUUUUUUCUACCUCU
Let7a RNA target N8	UUUUUUUUUU(5-LC-N-U)UUUUUUUACUACCUCU
Let7a RNA target N15	UUUUUUUUUU(5-LC-N-U)ACAACCUACUACCUCU
Let7a RNA target N19	UUUUUUUCUA(5-LC-N-U)ACAACCUACUACCUCU
U_{30}-biotin	[phos]UUUUUUUUUUUUUUUUUUUUUUUUUUUUUU[biotin]
DNA splint N6	AAAAAAAAAAAAGAGGTAGAAAAA
DNA splint N8	AAAAAAAAAAAAGAGGTAGTAAAA
DNA splint N15&N19	AAAAAAAAAAAAAAAAGAGGTAGTAGGTTGTAT
IVT DNA template t1U	CAAGCAGAAGACGGCATACGAGATAAAAAAGAGGTAAAAAAAAAAAAAAAAAAAAAATGATCGGAAGAGCGTCCCTATAGTGAGTCGTATTA
IVT DNA template t1A	CAAGCAGAAGACGGCATACGAGATAAAAAUGAGGTAAAAAAAAAAAAAAAAAAAAAATGATCGGAAGAGCGTCCCTATAGTGAGTCGTATTA
IVT DNA template t1G	CAAGCAGAAGACGGCATACGAGATAAAAACGAGGTAAAAAAAAAAAAAAAAAAAAAATGATCGGAAGAGCGTCCCTATAGTGAGTCGTATTA
IVT DNA template t1C	CAAGCAGAAGACGGCATACGAGATAAAAAGGAGGTAAAAAAAAAAAAAAAAAAAAAATGATCGGAAGAGCGTCCCTATAGTGAGTCGTATTA
IVT T7 promotor	TAATACGACTCACTATAGGG
Immobilization strand	[biotin]CAAGCAGAAGACGGCATACGAGAT
Visualizatiton strand	[Cy3-dT]GATCGGAAGAGCGTCCC

(5-LC-N-U) is 5-Aminohexylacrylamino-uridine, to which the fluorescent label is conjugated.

Table S4. Primers used for plasmid constructions for protein expression and purification in E. coli.

Primer name	Sequence	Notes
oPB198	aggttgtgaatgaacGccagacccttac	E623A RV
oPB199	tgggcattgCGgtttggcacgg	D585A FW
oPB200	gtaagggtctggCgttcattcacaacct	E623A FW
oPB201	ccgtgccaaacCGcaatgccca	D585A RV

Table S5. Primers used for plasmid construction and qPCR for RNA silencing in human cell lines.

a. Primers for plasmid construction

Name	Sequence (5′ to 3′)
pX linearization (FOR)	CACAGAGACATCTCAGGTAGCAC
pX linearization (REV)	AATTCGCCCCCTGCCCCGGCG
pmirGLO linearization (FOR)	TTCTAGTTGTTTAAACGAGCTCGCTAGCCTCGAGTCTAGA TACATACTTCTTTACATTCCAGTCGACCTGCAGGCATGCA AGCTGATATACATACTTCTTTACATTCCACCGGCTGCTAAC AAAGCCCGAAAGG
pmirGLO linearization (REV)	AGCGAGCTCGTTTAAACAACTAGAATTACACGGCG
pLKO.1 linearization (FOR)	TCTTGTGGAAAGGACGAAACACCGGGGAACTGCATGCA GACTGCCTGCTTGGGAAACATACTTCTTTATATGCCCATAT GGACCTGCTAAGCTATGGAATGTAAAGAAGTATGTATCTC AGGCCGGGACCTCTCTCGCCGCACTGATTTTTTTCCGC AGGTATGCACGCGTGAATTC
pLKO.1 linearization (REV)	CCGGTGTTTCGTCCTTTCCACAAG

b. Primers for qPCR.

Name	Sequence (5′ to 3′)
Fluc qPCR (FOR)	TCGTGCTGGAACACGGTAAA
Fluc qPCR (REV)	GTAACCTGGCTGGCCACATA
Rluc qPCR (FOR)	CAGCGACGATCTGCCTAAGA
Rluc qPCR (REV)	CCCTCGACAATAGCGTTGGA
Pri-miR-1-1 qPCR (FOR)	AGACTGCCTGCTTGGGAAAC
Pri-miR-1-1 qPCR (REV)	TCCATAGCTTAGCAGGTCCAT
U6 snRNA qPCR (FOR)	GTGCTCGCTTCGGCAGCAC
U6 snRNA qPCR (REV)	ATATGGAACGCTTCACGAATTTGCGTGTC

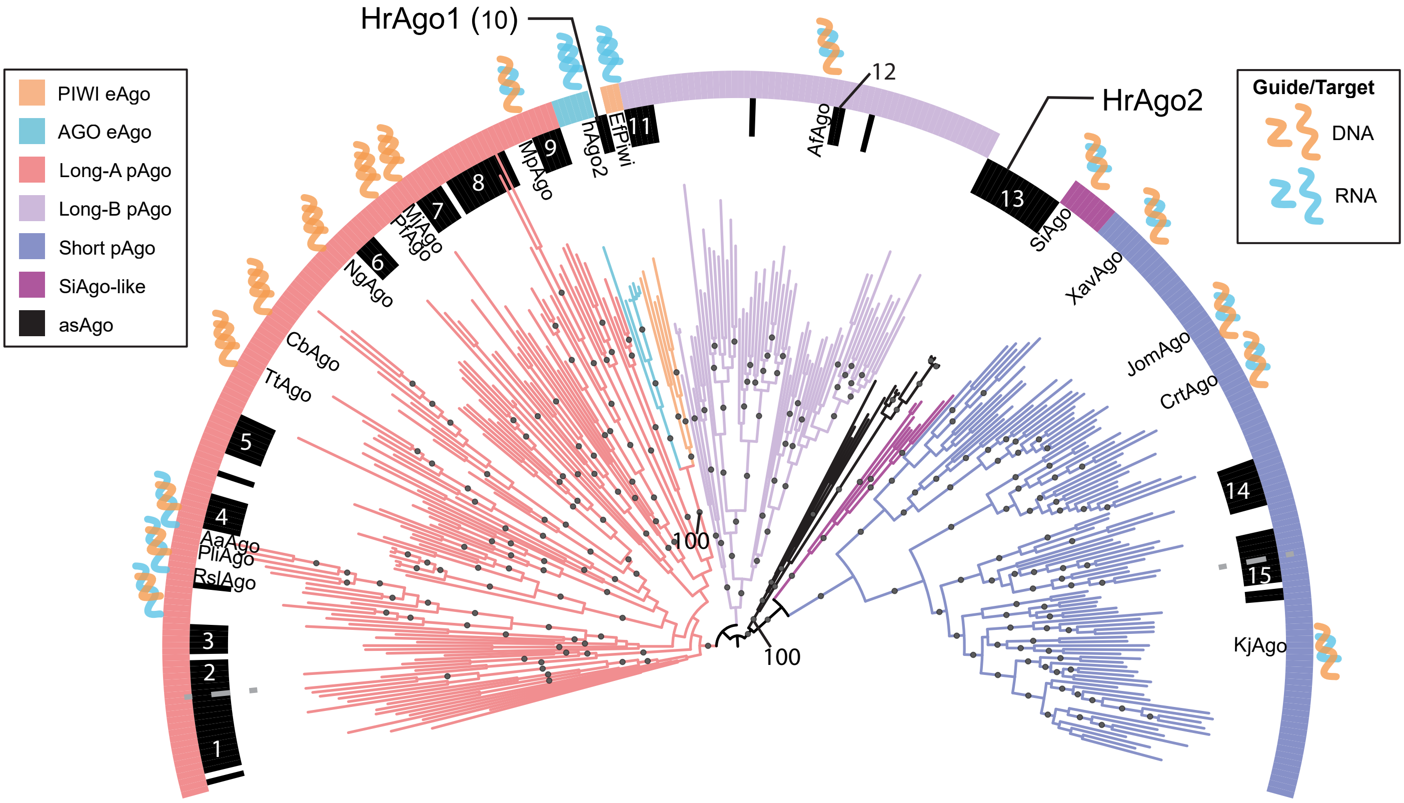

Fig. S1. Maximum-likelihood phylogenetic analysis of the MID-PIWI domains of Argonaute proteins showing that asAgos are polyphyletic (black pallets, subclades 1-15 denoted). 334 representative sequences and 572 sites were analyzed using IQ-tree based on Q.pfam+C60+F+G4 model. Different branch and ring colors indicate different major Argonaute clades. Various representative Argonautes (see Methods) and their primary guide/target preferences are indicated, while they may have secondary guide/target use. Ultrafast bootstrap 2 (UFBoot2) values above 95, calculated based on 1000 replicates, are shown in black circles. HrAgo1 and HrAgo2, and the UFBoot2 values at the base. of their respective clades are highlighted.

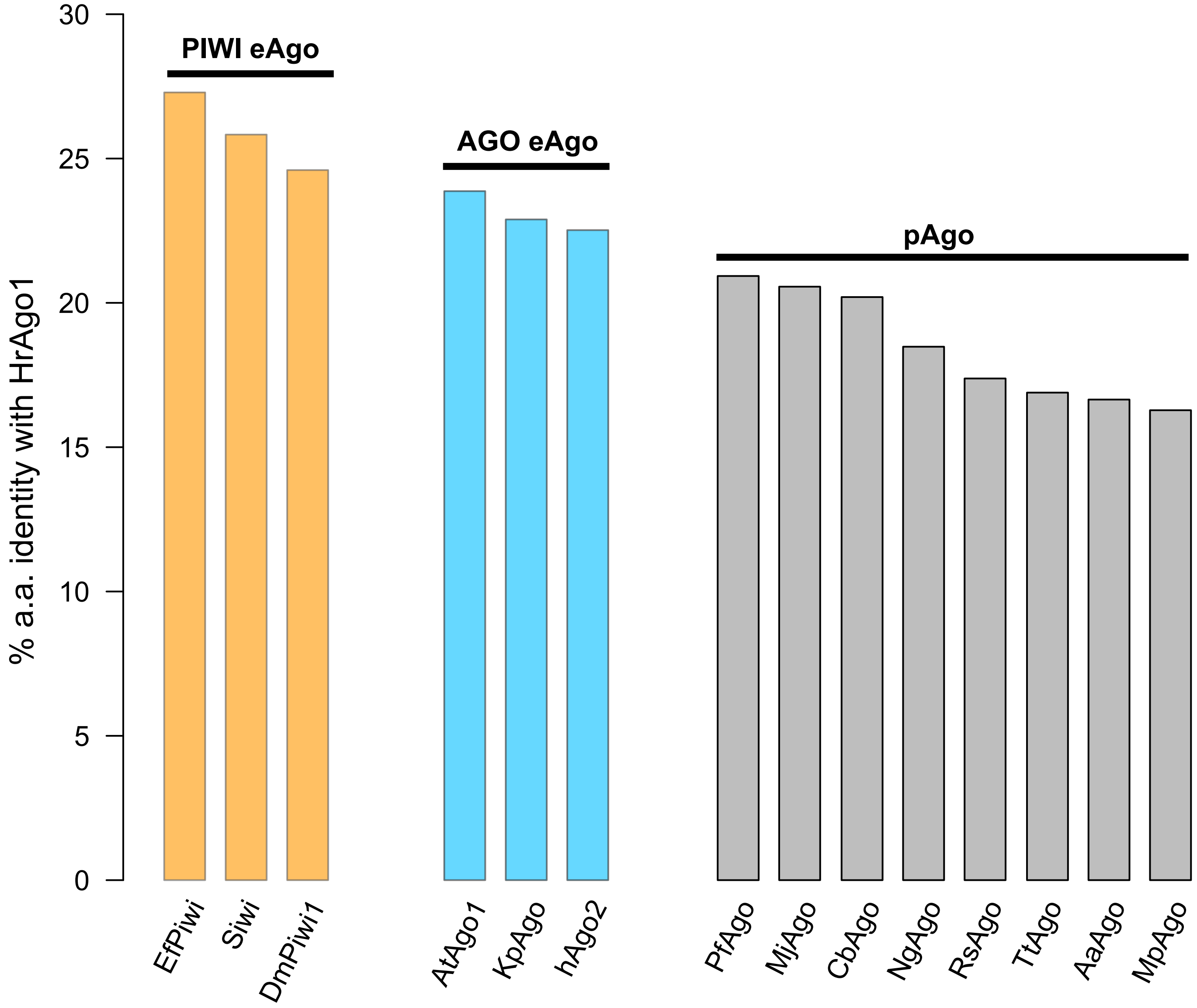

Fig. S2. Percentage amino acid identity conservation between HrAgo1 and various biochemically studied pAgos and eAgos. Source data are provided in the Source Data file.

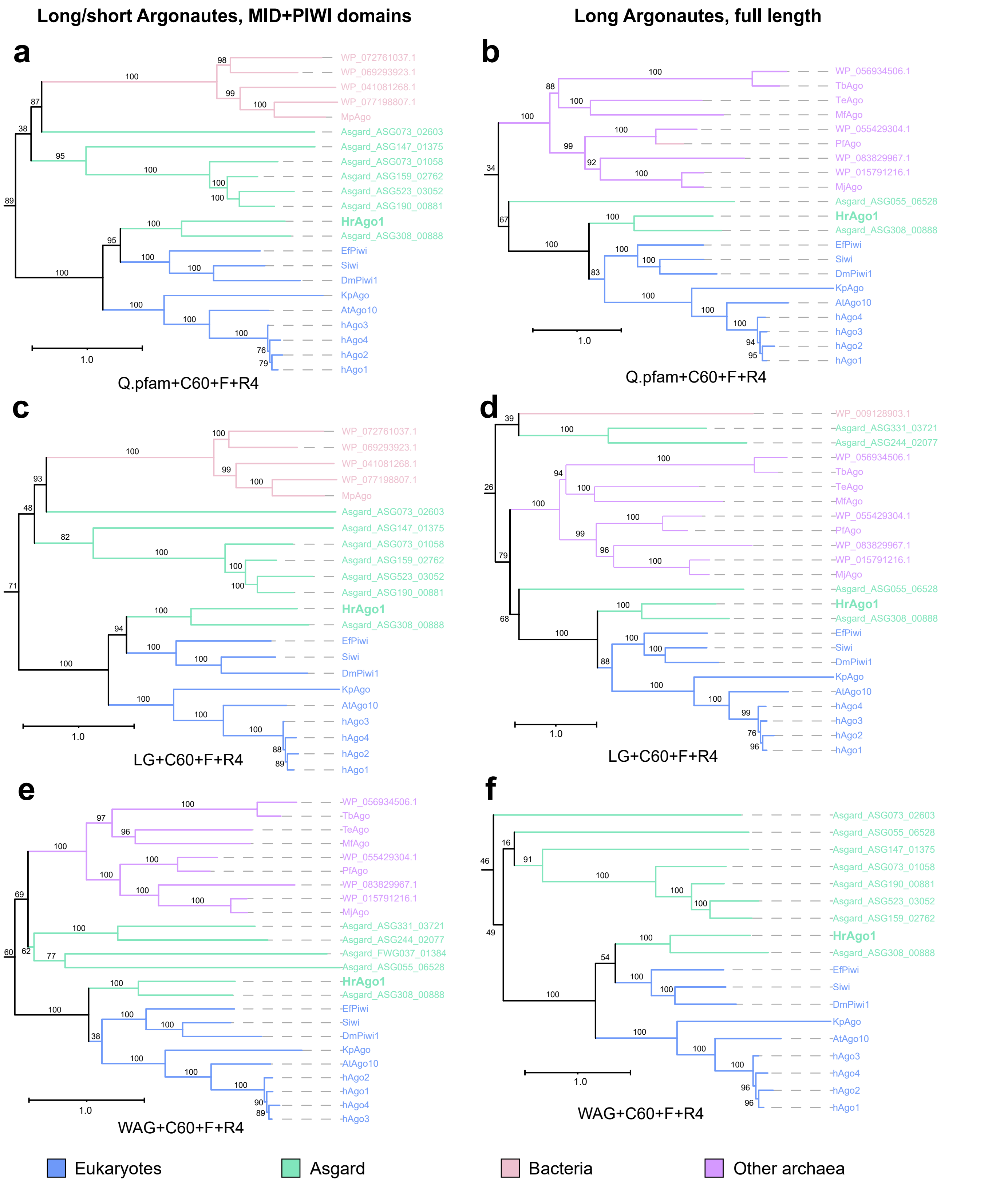

Fig. S3. Maximum-likelihood pAgo-asAgo-eAgo phylogenetic analyses support the close relation between HrAgo1 and eAgos, while the exact root of the eAgo clade is unstable. Legends are shown at the bottom. The analyses were done with the MID+PIWI domains of all Ago types (Left), and the full-length alignment of Long-A, Long-B, and the HrAgo2 clade (right), using three different kinds of mixture models under 1000 ultrafast bootstrap replicates in IQtree. Only branches close to the eAgo clade is shown. In **a, c,** and **f,** HrAgo1 is sister to the PIWI clade, while in **b, d,** and **e,** HrAgo1 is sister to the whole eAgo clade. This reflects a basal position that is difficult to resolve. Some other asAgos (in green) appeared basal to the eAgo-HrAgo1 clade in multiple conditions, but are supported by low bootstrap values. UFBoot2 values are shown.

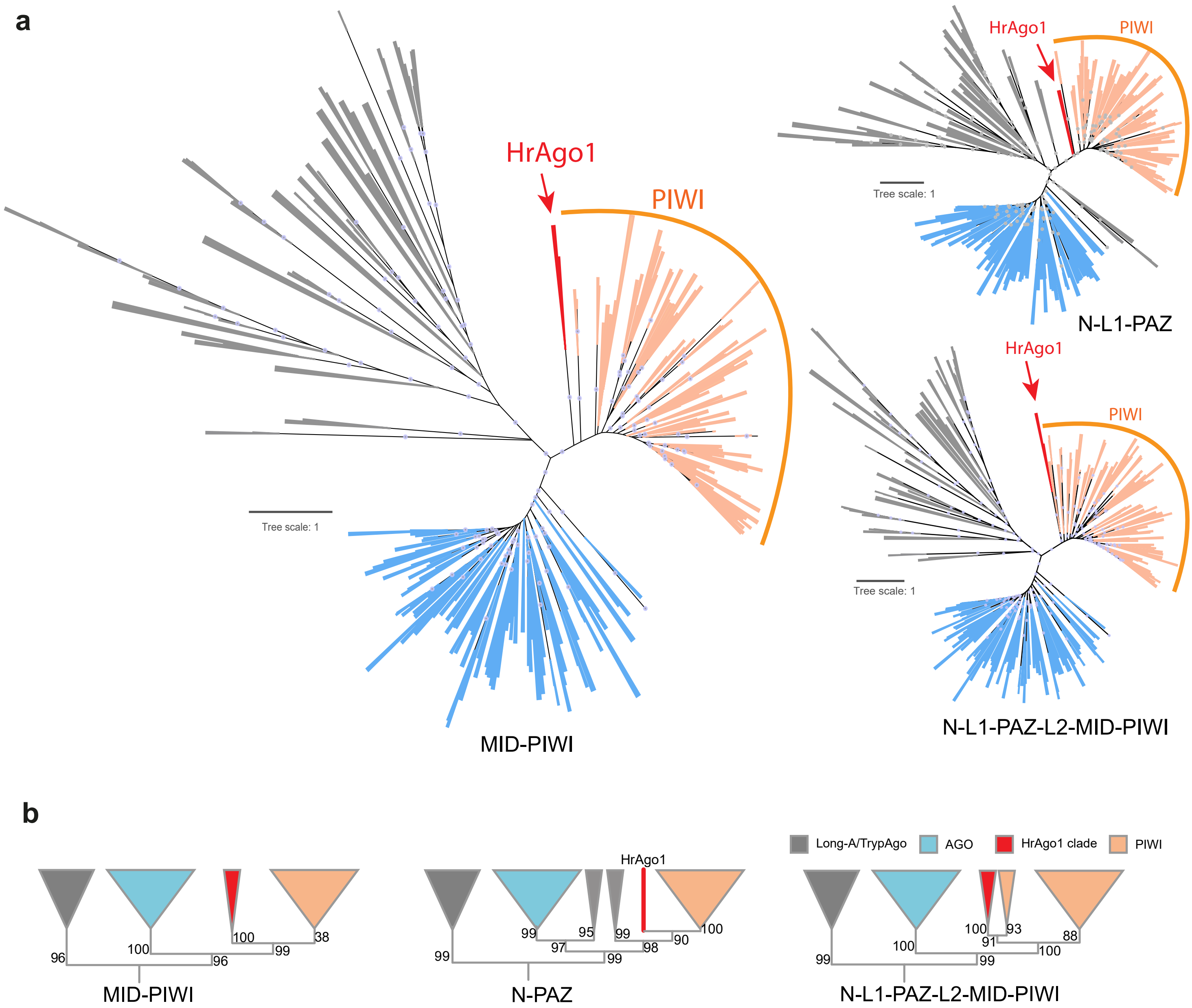

Fig. S4. Unrooted phylogenetic trees of different protein domain combinations showing that HrAgo1 is consistently positioned basal to the PIWI clade. The trees are shown in unrooted (**a**) and collapsed (**b**) formats. Grey, pAgo and TrypAgos. Blue, AGO clade. Orange, PIWI clade. Red, HrAgo1 clade. The domains used for phylogenetic analyses are indicated at the bottom of each tree. In a, grey circles indicate UFBoot2 values above 90. In b, bootstraps values are indicated.

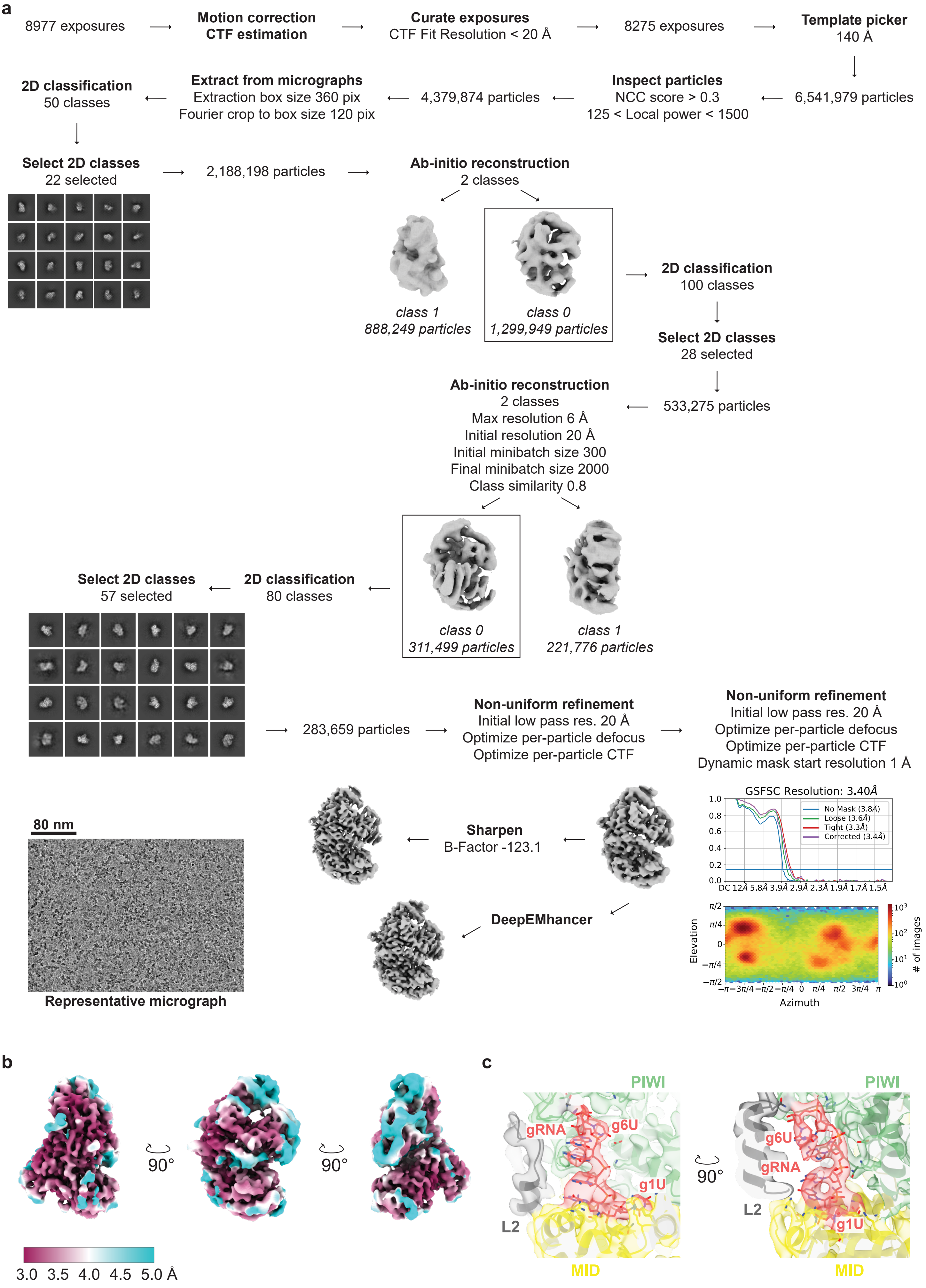

Fig. S5. Cryo-EM data processing for HrAgo1-gRNA complex. a, Cryo-EM image processing workflow for HrAgo1-gRNA. Unless specified, standard processing parameters were used. **b,** Cryo-EM densities of the HrAgo1-gRNA complex colored according to local resolution.

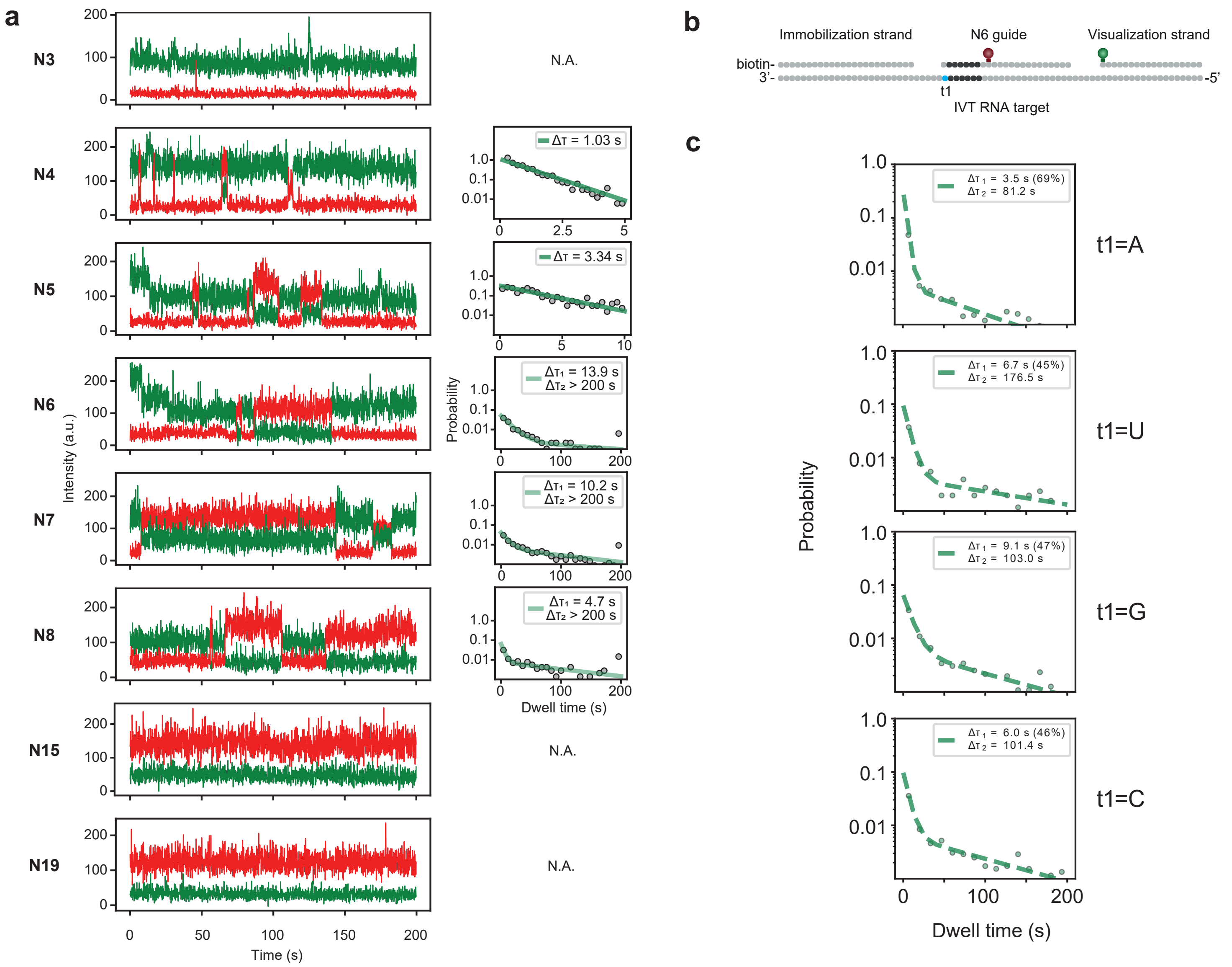

Fig. S6. Representative time traces and dwell time distributions with fit for HrAgo1. a, Representative time traces (left) and dwell time distributions with fit (right) for HrAgo1. Dwell time distributions were fit with a single or double exponential. N, match length between guide and target starting from the second nucleotide. N.A., no fit due to time resolution or observation time limit. Due to the observation time limit, the second dwell time is underestimated, therefore it is set to > 200 s for all match lengths that exhibit a stably bound population. For N3, the time resolution is limiting so the dwell time is set to < 0.1 s. **b,** Schematic of the construct used for the t1-target assays. **c,** Dwell time distributions with double exponential fit for targets with a different nucleotide at the first position (t1).

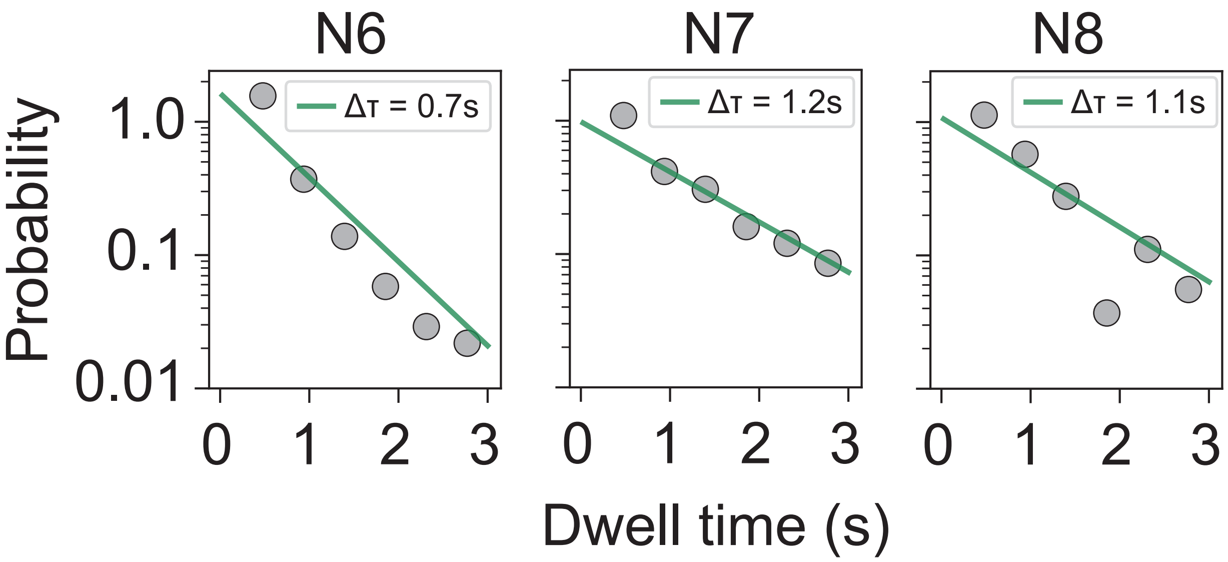

Fig. S7. Dwell time distributions with fit for EfPiwi. The dwell time distributions were fit with a single exponential. For shorter and longer match lengths, the time resolution (0.1s) and observation time (200s) were limiting so no fit could be made.

a

	Relative expression level (Pri-mir-1-1/U6 snRNA)
Parental *AGO1/2/3* KO cells	3.4E-06 (=0.00034%)
Puromycin-selected KO cells	5.0E-02 (=5%)

b

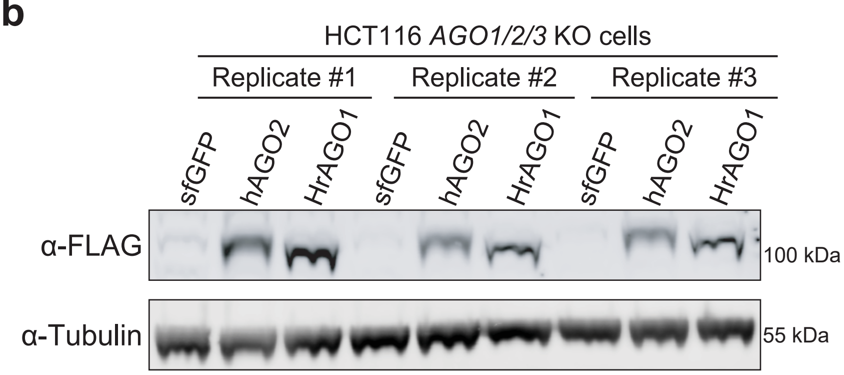

Fig. S8. Characterization of engineered human cell lines. a, qPCR results for pri-mir-1-1 from parental cells and puromycin-selected cells. Expression levels were normalized to U6 snRNA. **b,** Western blot results for ectopically expressed FLAG-hAGO2 and FLAG-HrAGO1. Tubulin was used as loading control. The uncropped gel images are provided in the Source Data file.
