# Supplementary material for: RNA-guided RNA silencing by an Asgard archaeal Argonaute
Source: Nat Commun. 2024 Jun 29;15:5499. doi: 10.1038/s41467-024-49452-1 (PMC11217426; doi:10.1038/s41467-024-49452-1)
Supplement: Supplementary file 3 — Description of Additional Supplementary Files [file 41467_2024_49452_MOESM3_ESM.pdf]

Description of Additional Supplementary Files:

Supplementary Dataset 1: A list of asAgos.

Supplementary Dataset 2: HMM profile of MID-PIWI for all Agos

Supplementary Dataset 3: HMM profile of full length eukaryotic AGO and some PIWI

Supplementary Dataset 4: HMM profile of full length eukaryotic PIWI and some AGO

Supplementary Dataset 5: Trimmed 334 MID-PIWI sequences

Supplementary Dataset 6: ML tree of 334 long/short argonaute MID-PIWI sequences as shown in Fig. S3c.

Supplementary Dataset 7: ML tree of 334 long/short argonaute MID-PIWI sequences as shown in Fig. 1a and S3a.

Supplementary Dataset 8: ML tree of 334 long/short argonaute MID-PIWI sequences as shown in Fig. S3e.

Supplementary Dataset 9: Trimmed 224 long argonaute sequences.

Supplementary Dataset 10: ML tree of 224 long argonaute sequences as shown in Fig. S3d.

Supplementary Dataset 11: ML tree of 224 long argonaute sequences as shown in Fig. S3b.

Supplementary Dataset 12: ML tree of 224 long argonaute sequences as shown in Fig. S3f.

Supplementary Dataset 13: Trimmed 264 MID-PIWI sequences.

Supplementary Dataset 14: ML tree of 264 MID-PIWI sequences as shown in Fig. S4.

Supplementary Dataset 15: Trimmed 264 N-PAZ-MID-PIWI sequences.

Supplementary Dataset 16: ML tree of 264 N-PAZ-MID-PIWI sequences as shown in Fig. S4.

Supplementary Dataset 17: Trimmed fasta file of 263 N-PAZ sequences.

Supplementary Dataset 18: ML tree of 263 N-PAZ sequences as shown in Fig. S4.
