# Supplementary material for: RNA-guided RNA silencing by an Asgard archaeal Argonaute
Source: Nat Commun. 2024 Jun 29;15:5499. doi: 10.1038/s41467-024-49452-1 (PMC11217426; doi:10.1038/s41467-024-49452-1)
Supplement: Supplementary file 2 — Peer Review File [file 41467_2024_49452_MOESM2_ESM.pdf]

nature portfolio

Peer Review File

RNA-guided RNA silencing by an Asgard archaeal Argonaute

Open Access This file is licensed under a Creative Commons Attribution 4.0 International License, which permits use, sharing, adaptation, distribution and reproduction in any medium or format, as long as you give appropriate credit to the original author(s) and the source, provide a link to the Creative Commons license, and indicate if changes were made. In the cases where the authors are anonymous, such as is the case for the reports of anonymous peer reviewers, author attribution should be to 'Anonymous Referee' followed by a clear attribution to the source work. The images or other third party material in this file are included in the article's Creative Commons license, unless indicated otherwise in a credit line to the material. If material is not included in the article's Creative Commons license and your intended use is not permitted by statutory regulation or exceeds the permitted use, you will need to obtain permission directly from the copyright holder. To view a copy of this license, visit http://creativecommons.org/licenses/by/4.0/.

Editorial Note: This manuscript has been previously reviewed at another journal that is not operating a transparent peer review scheme. This document only contains reviewer comments and rebuttal letters for versions considered at *Nature Communications*.

Reviewer #1 (Remarks to the Author):

I am satisfied with the revision and have no further concerns.

Reviewer #2 (Remarks to the Author):

The authors have adequately addressed all my questions and concerns. I found only one small error in wording in one of the newly introduced sentences. It should read:

"HrAgo1 clusters with and has the same conserved domains as eAgos ..."

After correcting this sentence, the manuscript is ready for publication.

The responses are in blue.

Reviewer #1 (Remarks to the Author):

I am satisfied with the revision and have no further concerns.

We thank the reviewer for the thorough review of our paper.

Reviewer #2 (Remarks to the Author):

The authors have adequately addressed all my questions and concerns. I found only one small error in wording in one of the newly introduced sentences. It should read:

"HrAgo1 clusters with and has the same conserved domains as eAgos ..."

After correcting this sentence, the manuscript is ready for publication.

We thank the reviewer for the thorough review of our paper. The sentence is now corrected.
